# Predicting diagnostic gene biomarkers associated with immune infiltration in patients with diabetes

**DOI:** 10.1590/1414-431X2026e15577

**Published:** 2026-09-07

**Authors:** Juan Feng, Tianhang Zhao, Sien Lai, Yiyang Huang, Yufen Xie, Guojun Chen

**Affiliations:** 1 The Third Affiliated Hospital, School of Medicine, Foshan University, Foshan, China; 2 Guangdong Provincial Engineering and Technology Research Center for Gene Editing, School of Medicine, Foshan University, Foshan, China; 3 Department of Emergency, First People's Hospital of Foshan, (Foshan Hospital Affiliated to Southern University of Science and Technology), School of Medicine, Southern University of Science and Technology, Guangdong, China

**Keywords:** Diabetes, Diagnostic, Biomarkers, Immune infiltration, Prediction

## Abstract

Diabetes is a global public health problem with various complications, which can lead to disability and mortality. This study identified potential diagnostic markers for diabetes and explored the immunometabolic mechanisms in the pathological process. The gene expression of 17 diabetes cases and 16 normal controls were obtained from the Gene Expression Omnibus (GEO) database. The “limma” package was employed for screening differentially expressed genes (DEGs). Gene functions and enriched pathways of DEGs were analyzed via Gene Ontology (GO) and Kyoto Encyclopedia of Genes and Genomes (KEGG) enrichment analyses. Candidate key genes were screened using the least absolute shrinkage and selection operator (LASSO) regression model and support vector machine recursive feature elimination (SVM-RFE) analysis. The diagnostic effectiveness of identified markers was further verified via the receiver operating characteristic (ROC) curve. The compositional patterns of immune cell infiltration and signaling pathway enrichment associated with key genes were explored via single sample Gene Set Enrichment Analysis (ssGSEA) and GSEA analysis, respectively. Possible miRNAs interacting with key genes were predicted via miRcode database. *B2M*, *FTL*, *SH3BGRL3*, and *SOD2* were recognized as diagnostic markers for diabetes based on LASSO regression and the support vector machine recursive feature elimination (SVM-RFE) feature selection algorithm. Analysis of immune cell infiltration demonstrated that the four key genes were related to B cells, neutrophils, macrophages, and CD8+ T cells. The diagnostic value of *B2M*, *FTL*, and *SOD2* for diabetes was higher than that of *SH3BGRL3* according to the ROC curve. Validation experiments indicated that the mRNA expression of *B2M* and *FTL* was increased in liver tissues of diabetic mice. *B2M* and *FTL* can act as diagnostic markers for diabetes and contribute to new understandings of the disease's molecular mechanisms.

## Introduction

Diabetes is a common public health problem worldwide, which is usually accompanied by multiple complications, including macrovascular, microvascular, and neurologic complications. The macrovascular complications of diabetes affect the heart, brain, and legs, with atherosclerosis of the coronary arteries being the main cause of death in diabetic patients (1,2). The microvascular complications of diabetes primarily affect the eyes and kidneys (1). Diabetic retinopathy is the leading cause of blindness among patients with diabetes, and diabetic nephropathy is the most common cause of end-stage renal disease in these patients (3). Therefore, the disability and mortality caused by diabetes and its complications contribute to a significant individual and social burden worldwide. However, studies on the pathogenesis and early diagnosis of diabetes are still limited.

The interaction between environmental and genetic factors initiates or accelerates the disease process of diabetes (4), although the possible underlying mechanisms are complex and not fully understood. Integrating knowledge of genetics, immunology, and metabolism can improve the precise prediction and treatment of diabetes. Growing evidence suggests that many candidate genes are closely related to diabetes and its associated complications, such as *VEGF* for retinopathy, *ADIPOQ* for coronary artery disease, and *ELMO1* for nephropathy (4,5). Research on pharmacogenetics can help enable precise treatment for diabetes. Variations in the *TCF7L2* locus and genes encoding the sulfonylurea receptor, such as *KCNJ11* and *ABCC8*, may increase the risk of type 2 diabetes and influence the metabolism and pharmaceutical effects of sulfonylurea drugs (6). Mutations in key genes associated with pancreatic development (such as *PTF1A*, *GATA6*, and *PDX1*) may lead to pancreatic dysplasia and diabetes (4). Therefore, the pathogenesis, prognosis, and precise treatment of diabetes are closely related to genetic factors.

Immune cell infiltration plays an important role in the occurrence and development of diabetes. Autoantigens and immune cell infiltration in the pancreatic islets are the main causes of autoimmune-mediated β-cell dysfunction (7-9). Macrophages, CD4+ T cells, CD8+ T cells, and Tregs have been shown to affect the progression of diabetes (8-10), providing new insights into the significance of immune modulation in diabetes. Therefore, identifying novel biomarkers and targeting these immune mechanisms are needed to develop future early diagnostic and therapeutic approaches for diabetes.

Microarray technology combined with integrated bioinformatics analysis has been used to screen key genes that play an important role in the diagnosis and prognosis evaluation of many diseases (11-14). In this study, two gene chip datasets were downloaded from the Gene Expression Omnibus (GEO) database (GSE23561 and GSE15932) and merged into a meta-data cohort. Differentially expressed genes (DEGs) were analyzed between the diabetes and control groups. Gene function and pathway enrichment analyses of DEGs were performed using bioinformatics methods. Machine learning algorithms were applied to screen and identify diagnostic biomarkers of diabetes. In addition, the diagnostic effectiveness of the identified markers was further verified using receiver operating characteristic (ROC) curve analysis in the GSE20966 dataset. The mRNA expression of key genes was also validated in diabetic animal samples. Immune cell infiltration and signaling pathway enrichment associated with key genes were estimated based on the merged cohorts using single sample Gene Set Enrichment Analysis (ssGSEA) and GSEA analyses, respectively. The relationship between the identified biomarkers and infiltrating immune cells was explored to provide a basis for further research. Furthermore, the metabolic pathways associated with diabetes and the possible miRNAs interacting with the key genes were predicted using Gene Set Variation Analysis (GSVA) and the miRcode database, respectively, which also lay the foundation for understanding the metabolic and molecular mechanisms of diabetes.

## Material and Methods

### Gene expression data acquisition

The GSE23561 data file was downloaded from the NCBI GEO public database and annotated with GPL10775 as a Series Matrix File. The file contains data from 17 groups of expression profiles (9 normal groups and 8 diabetic groups). In addition, the GSE15932 Series Matrix File was obtained from the GEO public database, annotated on the GPL570 platform, and consists of 16 groups of expression profile data (8 normal groups and 8 diabetic groups). The GSE20966 dataset, collected from beta cells and containing 10 diabetic samples and 10 control samples, was used as the validation cohort with the Affymetrix Human X3P Array.

### Data processing and DEGs analysis

The two datasets were combined, and data correction between chips was performed using the Combat algorithm to eliminate batch effects (14). The DEGs between the 16 diabetic samples and 17 control samples were identified using the “limma” package (http://www.bioconductor.org/) (14). The screening criteria for DEGs were P-value >0.05 and |logFC|>0.585.

### Gene ontology and KEGG enrichment analysis

To study the biological functions and signaling pathways involved in the occurrence and development of diabetes mellitus, the Metascape database (www.metascape.org) was used to annotate and visualize DEGs for GO analysis and KEGG pathway analysis. A minimum overlap of ≥3 and P≤0.01 were considered statistically significant.

### Exploration of potential diagnostic biomarkers

The least absolute shrinkage and selection operator (LASSO) logistic regression and support vector machine recursive feature elimination (SVM-RFE) algorithms were used to select characteristic diagnostic markers of disease (15). The LASSO regression algorithm was implemented with the “glmnet” package in R to identify feature genes associated with diabetes (16). SVM-RFE, a machine learning method based on support vector machines, identifies the best variables by removing feature vectors generated by SVM (15). SVM-RFE was performed to select appropriate feature genes using the “e1071” R package (16). The overlapping genes identified by both algorithms were considered potential diagnostic genes for diabetes.

### Evaluation and validation of the diagnostic value of biomarkers for diabetes

The predictive value of the screened biomarkers was validated by generating a ROC curve using mRNA expression data from the GSE20966 dataset. The area under the ROC curve (AUC) was used to evaluate diagnostic performance in distinguishing diabetes from control samples. In addition, we conducted experimental verification with diabetic animal samples previously collected. Total RNA from the livers of control and diabetic mice was extracted using TRIzol reagent (CWBIO, China). One microgram of total RNA was reverse-transcribed into cDNA with the StarScript III All-in-one RT Mix with gDNA Remover kit (GenStar, China). The primer sequences for *B2M* were forward primer 5′-ACCGTCTACTGGGATCGAGA-3′ and reverse primer 5′-TGCTATTTCTTTCTGCGTGCAT-3′. The primer sequences for *FTL* were forward primer 5′-CGCGGACCCTCATCTCT-3′ and reverse primer 5′-CTGCGCTGGTTGTGGC-3′. The mRNA expression of key genes was normalized to β-actin.

### Analysis of immune cell infiltration

The ssGSEA was used to explore the infiltration of different immune cell types, immune-related functions, and immune-related pathways in diabetic and control groups using the R package “GSVA” (17). Differences in immune cell infiltration between diabetic and control samples were visualized using violin plots generated with the R package “vioplot” (14). The R package “corrplot” was used for correlation analysis and visualization of these infiltrating immune cells (14). The relationship between the identified biomarkers and immune cell infiltration levels was examined using Spearman's rank correlation analysis, and the results were visualized with the R package “ggplot2” (14).

### Gene set variation analysis

Gene set variation analysis (GSVA) is a nonparametric, unsupervised method for evaluating transcriptome gene set enrichment. By comprehensively scoring the gene set of interest, GSVA converts gene-level changes into pathway-level changes, allowing assessment of the biological function of each sample. In this study, gene sets were downloaded from the Molecular Signatures Database (v7.0), and the GSVA algorithm was used to score each gene set to evaluate potential biological function changes in different samples. In addition, metabolism-associated pathways in the diabetic and control groups were analyzed using the R package “GSVA”.

### Analysis of possible miRNAs interacting with key genes

The possible miRNAs interacting with the key genes were predicted using the miRcode database. In addition, the interaction network of miRNAs and target key genes was visualized with Cytoscape software.

### Statistical analysis

Statistical analysis was performed using R (version 4.0). All statistical tests were two-sided, and P<0.05 was considered statistically significant.

## Results

### Identification of DEGs between diabetes and control groups

**Figure 1:**
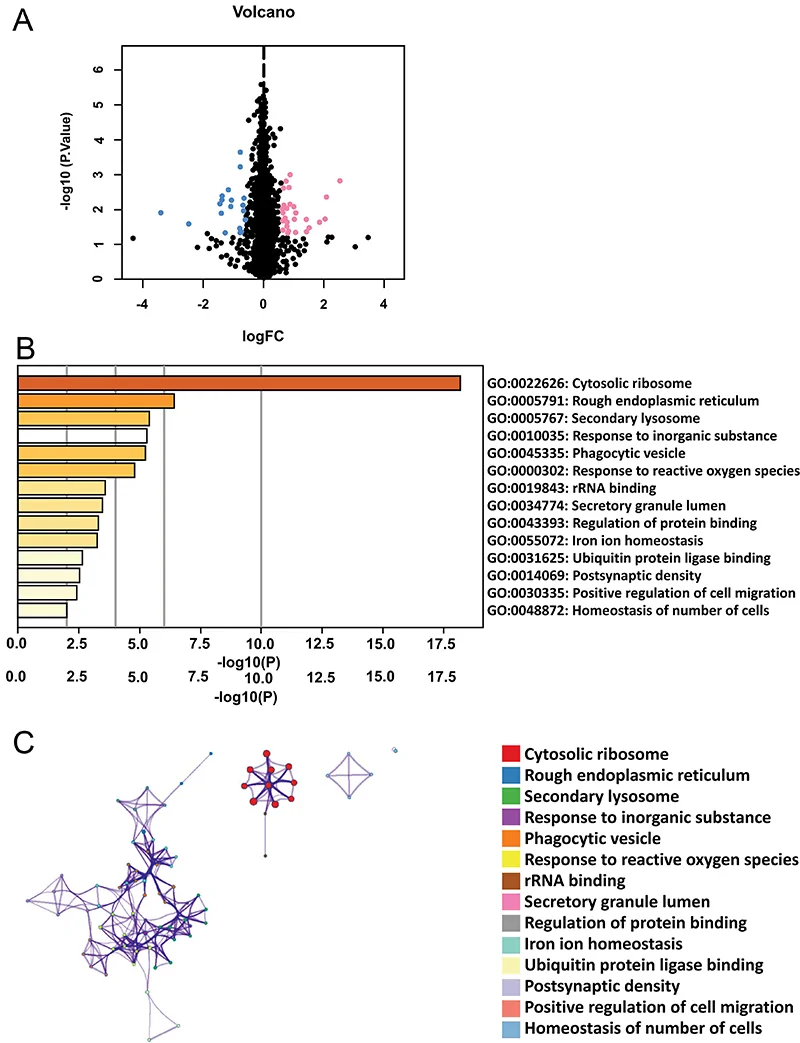
**A**, Screened differentially expressed genes between diabetic and control group according to gene chip data. **B**, Gene Ontology (GO) enrichment analysis of differentially expressed genes. **C**, KEGG pathway analysis of differentially expressed genes.

In this study, data from 17 control and 16 diabetic samples across two GEO datasets (GSE23561 and GSE15932) were analyzed retrospectively. The “limma” package was used to identify the DEGs in the groups after batch effects were removed. Batch effects were eliminated using the Combat algorithm (Supplementary Figure S1A and B). The analysis identified 56 DEGs, including 33 up-regulated genes and 23 down-regulated genes (Figure 1A). The expression of these DEGs is shown in Supplementary Table S1.

### GO and KEGG pathway analyses of DEGs

The function and pathway of DEGs were analyzed via GO and KEGG pathway enrichment analyses. The DEGs were mainly enriched in the cytosolic ribosome, rough endoplasmic reticulum, and secondary lysosome and other pathways (Figure 1B and C).

### Identification of potential diagnostic biomarkers

**Figure 2:**
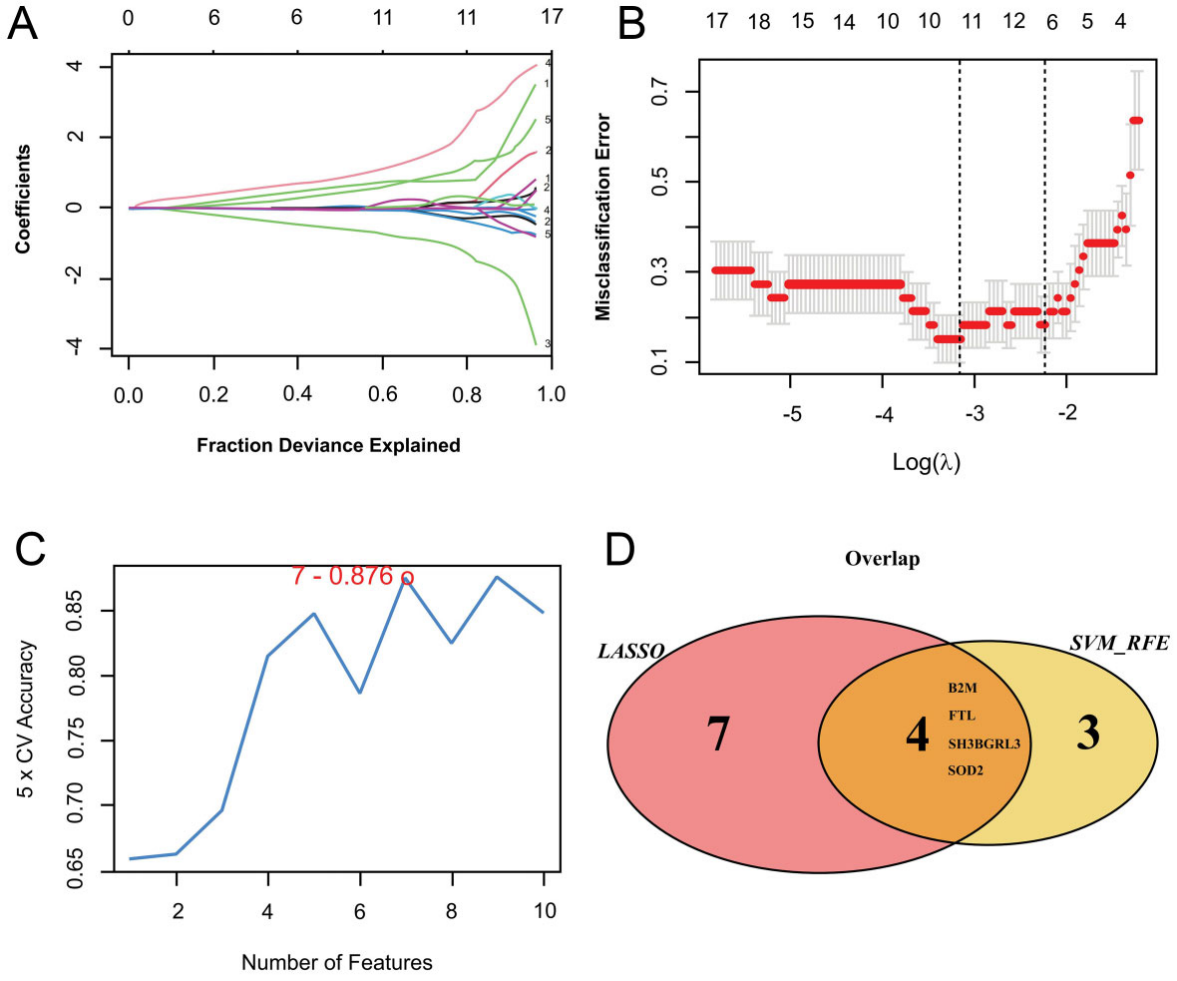
Screened feature biomarker genes in diabetes via combination use of least absolute shrinkage and selection operator (LASSO) regression and support vector machine-recursive feature elimination (SVM-RFE) algorithm. **A** and **B**, Characteristic genes of diabetes identified via LASSO regression. **C**, Plot of the characteristic genes of diabetes identified via SVM algorithm. **D**, Overlapping genes between LASSO regression and SVM algorithm.

To further explore the key genes affecting diabetes, we combined LASSO regression and the SVM-RFE feature selection algorithm to identify characteristic genes associated with diabetes. A total of 11 genes were identified as characteristic genes of diabetes by Lasso regression (Figure 2A and B). On the other hand, the top 7 key genes with the highest accuracy were screened with the SVM-RFE algorithm to evaluate the characteristic genes in diabetes (Figure 2C). Four feature genes (*B2M*, *FTL*, *SH3BGRL3*, and *SOD2*) overlapped between the two algorithms and were ultimately selected (Figure 2D). These genes will serve as candidate key genes for subsequent research.

### Evaluation and validation of the diagnostic efficacy of biomarkers in diabetes

**Figure 3:**
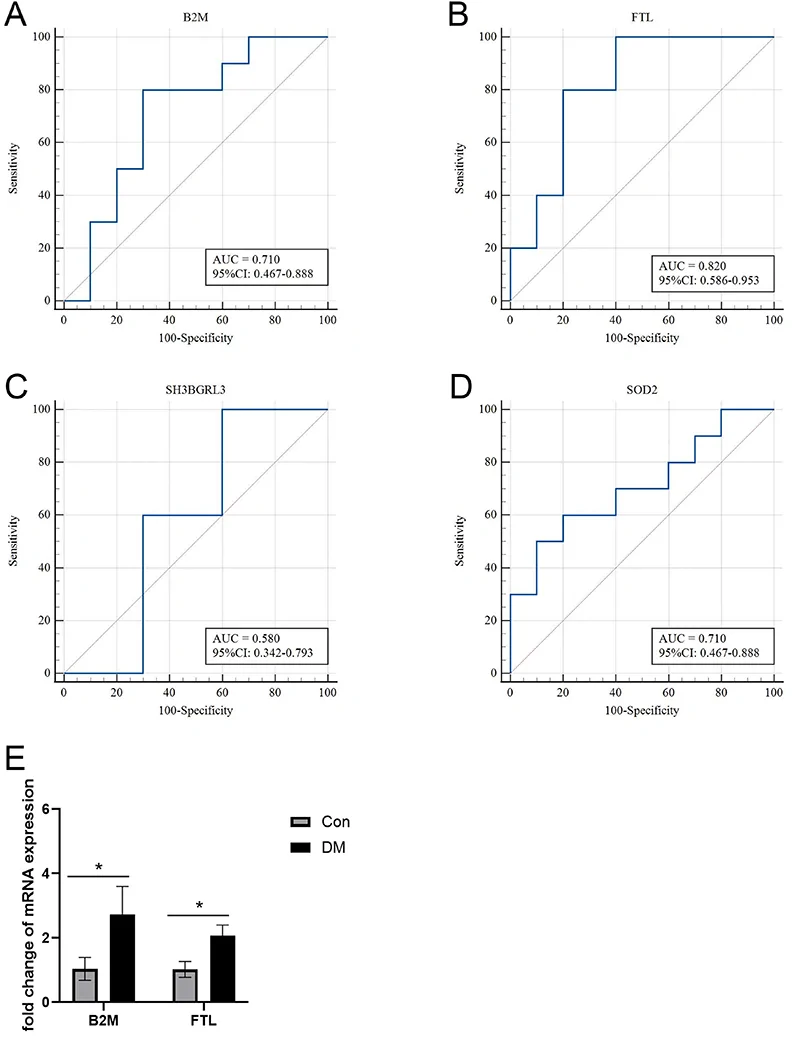
ROC curve and mRNA expression of the identified markers. ROC curve of B2M (**A**), FTL (**B**), SH3BGRL3 (**C**), and SOD2 (**D**) in the GSE20966 dataset. mRNA expression of B2M (**E**) and FTL (**E**) in liver tissues of control (Con) and diabetic mice (DM). Data are reported as means and SD (n=4). *P<0.05; Student's *t*-test.

The four biomarkers demonstrated diagnostic value in diabetes. The AUC of *B2M*, *FTL*, *SH3BGRL3*, and *SOD2* were 0.710 (95%CI: 0.467-0.888), 0.820 (95%CI: 0.586-0.953), 0.580 (95%CI: 0.342-0.793), and 0.710 (95%CI: 0.467-0.888), respectively (Figure 3A-D). The clinical characteristics of control and type 2 diabetic subjects in the validation cohort (GSE20966) are shown in Supplementary Table S2. These results indicated that *B2M*, *FTL*, and *SOD2* have greater diagnostic value for diabetes than *SH3BGRL3*. Furthermore, verification experiments showed that mRNA expression of *B2M* and *FTL* was increased in the liver tissues of diabetic mice (Figure 3E).

### Analysis of immune cell infiltration

**Figure 4:**
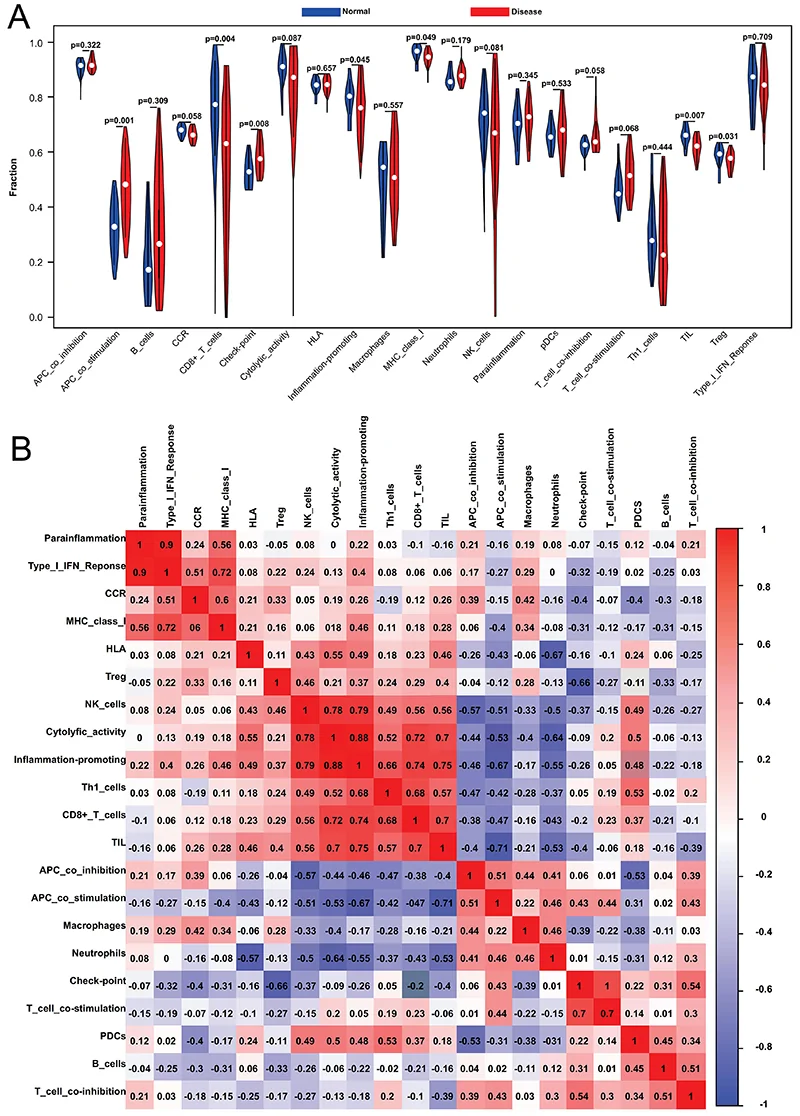
Distribution and visualization of immune cell infiltration. **A**, Comparison of 21 immune cell subtypes between diabetic and normal tissues. Blue and red colors represent normal and diabetes samples, respectively. **B**, Correlation matrix of all 21 immune cell subtype compositions. The horizontal and vertical axes indicate immune cell subtypes. Higher, lower, and no correlation levels are displayed in red, blue, and white, respectively.

**Figure 5:**
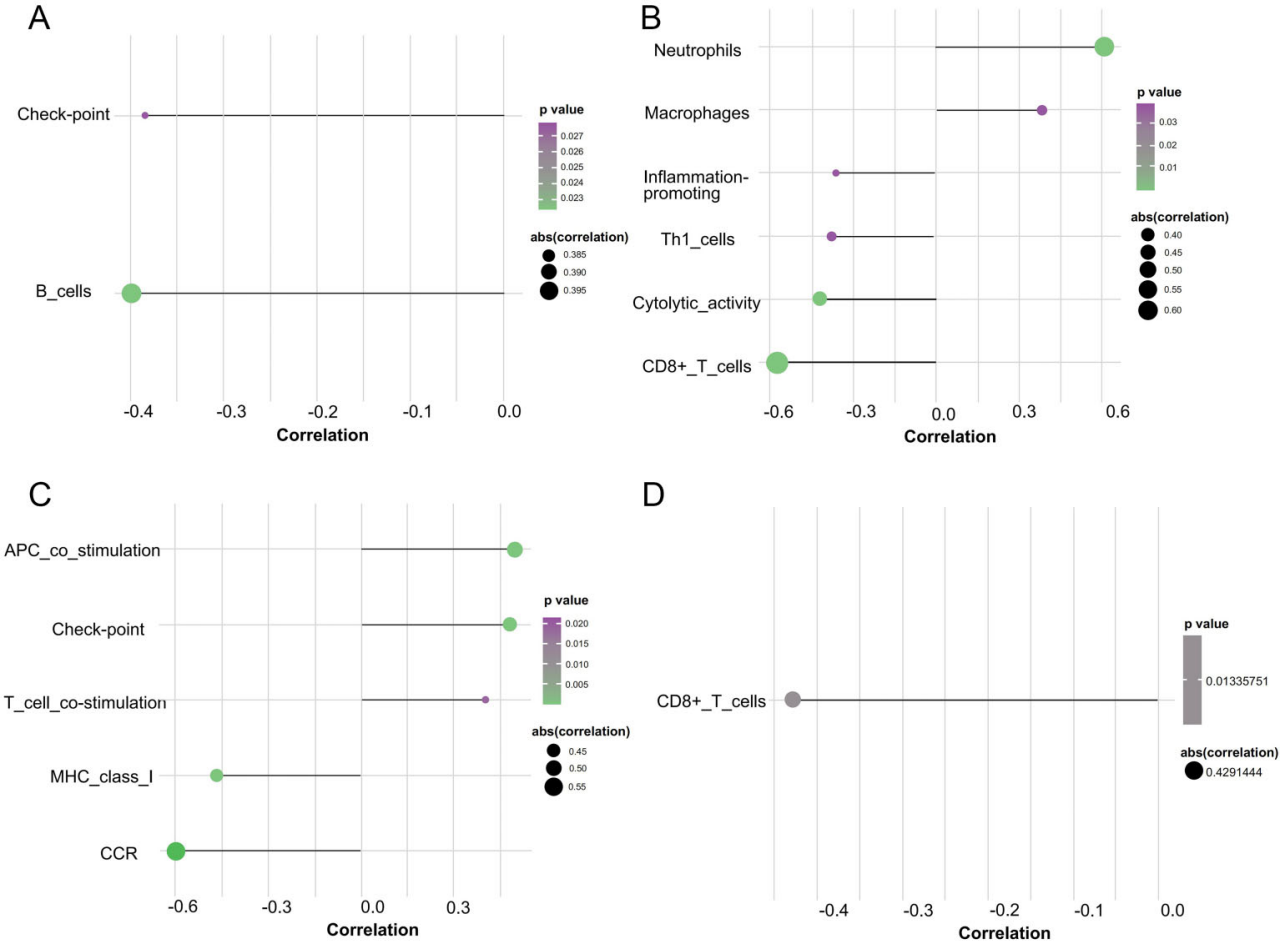
Correlations between *B2M* (**A**), *FTL* (**B**), *SH3BGRL3* (**C**), *SOD2* (**D**), and infiltrating immune cells in diabetes.

The immune microenvironment is primarily composed of immune-related fibroblasts, immune cells, extracellular matrix, various growth factors, and inflammatory factors, all of which play important roles in the diagnosis, prognosis, and clinical treatment sensitivity of diseases. The antigen presenting cell (APC) co-stimulation and check-point levels in the disease group were significantly higher than those in normal patients (Figure 4A). There were multiple significant correlations among the levels of immune cell infiltration (Figure 4B). We further explored the relationships between key genes and immune cells (Supplementary Tables S3-6). *B2M* was negatively correlated with B cells and check-point (Figure 5A). *FTL* was positively correlated with neutrophils and macrophages, and negatively correlated with CD8+ T cells, cytolytic activity, Th1 cells, and proinflammatory function (Figure 5B). *SH3BGRL3* was positively correlated with APC co-stimulation, check-point, and T cell co-stimulation, and negatively correlated with CCR and MHC class I (Figure 5C). SOD2 was negatively correlated with CD8+ T cells (Figure 5D).

### Specific signaling pathways enrichment analysis of key genes in diabetes

**Figure 6:**
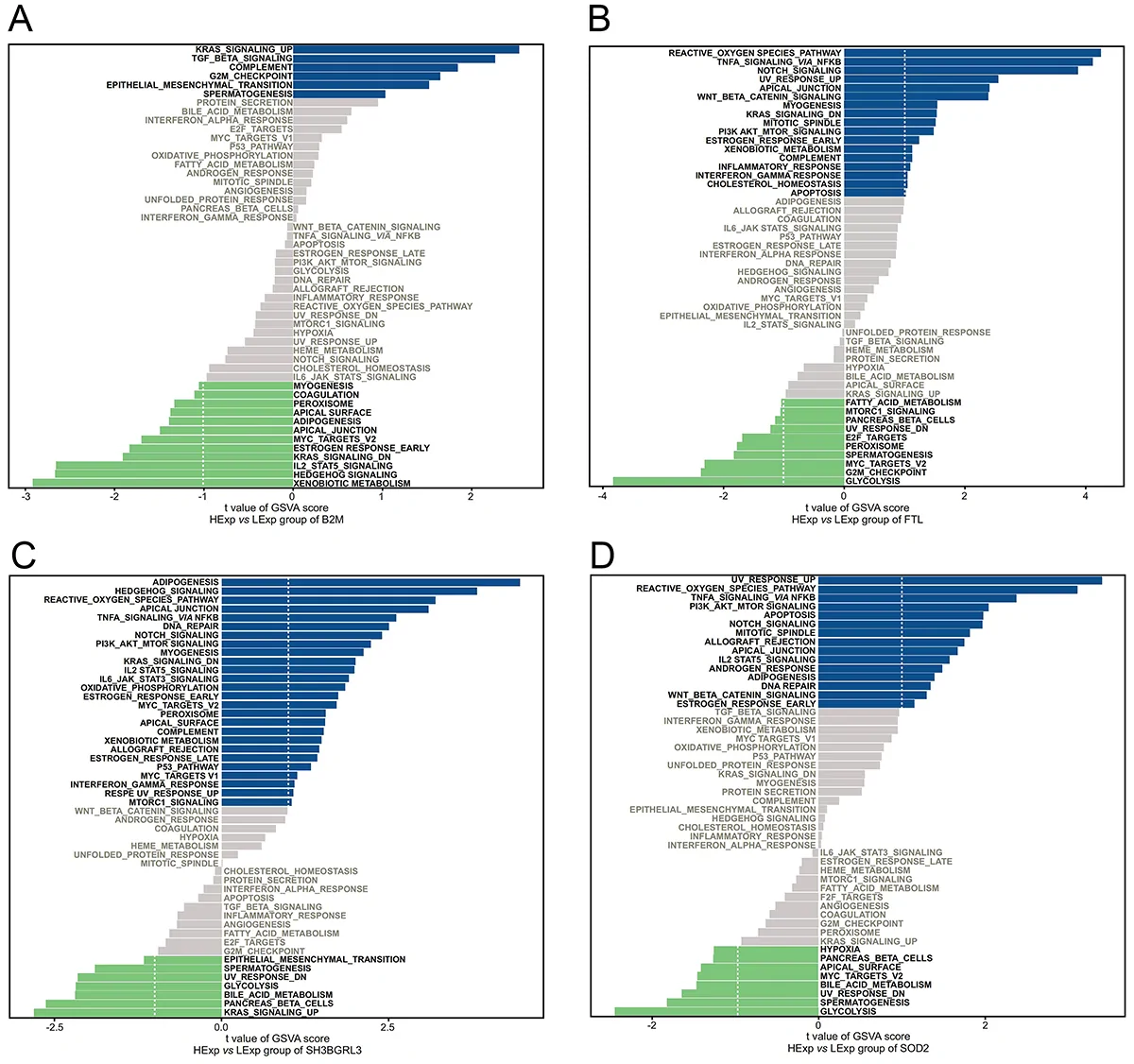
Signaling pathways enrichment analysis of *B2M* (**A**), *FTL* (**B**), *SH3BGRL3* (**C**), and *SOD2* (**D**) via gene set variation analysis (GSVA).

We further investigated the specific signaling pathways involved in the screened key genes to explore the potential molecular mechanisms by which these genes affect disease progression. The *B2M* gene is enriched in KRAS signaling, TGFβ signaling, complement, and other pathways (Figure 6A). The *FTL* gene is enriched in the reactive oxygen species pathway, TGFA signaling pathway, Notch signaling pathway, and others (Figure 6B). The *SH3BGRL3* gene is enriched in adipogenesis, Hedgehog signaling pathway, reactive oxygen species pathway, and others (Figure 6C). The *SOD2* gene is enriched in UV response, reactive oxygen species pathway, TNFA signaling pathway, PI3K-AKT-mTOR signaling, apoptosis, and others (Figure 6D).

### Analysis of metabolic pathway associated with diabetes

**Figure 7:**
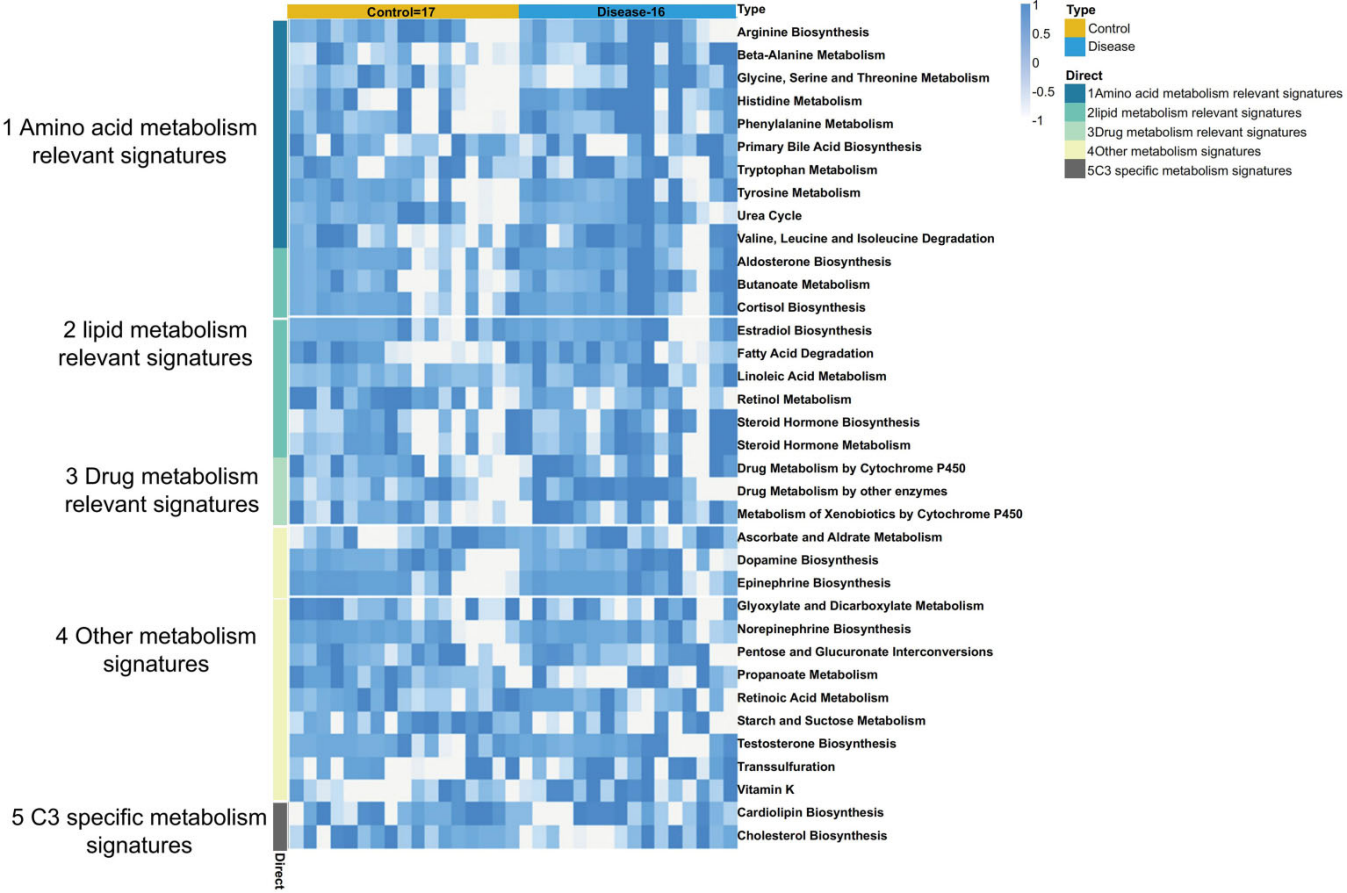
Possible metabolic pathways between diabetic and control groups.

The metabolic pathway analysis between the diabetic and control groups is shown in the metabolic pathway heat map (Figure 7). The scores of the diabetes samples in the amino acid metabolism relevant signature classification were higher than those of the normal samples.

### Analysis of miRNAs interacting with the key genes

The four key genes were differentially expressed in people with diabetes compared to controls, although the mechanism underlying this differential expression remained unclear. Since miRNAs can post-transcriptionally regulate gene expression by binding to target mRNAs, we further explored the miRNAs interacting with these key genes using the miRcode database. A total of 71 miRNAs and 117 mRNA-miRNA interaction pairs were predicted (Supplementary Figure S2).

## Discussion

Diabetes has become prevalent worldwide, leading to numerous microvascular and macrovascular complications that cause mortality and disability in diabetic patients if early diagnosis and treatment are not performed. Therefore, it is urgent to find solutions to address the health problem of diabetes and its associated complications.

Diabetes is a complex disorder associated with multiple genetic and environmental factors (7). Genetic factors, immunity, metabolism, inflammatory responses, and intestinal flora are involved in the onset and progression of diabetes. Mutations in *HNF1A*, *GCK*, *KCNJ11,* and *ABCC8* genes are closely associated with monogenic diabetes (18-21). Genetic variations affecting the pharmacokinetics and efficacy of diabetic drugs, such as *SLC22A1*, *SLC22A2*, *SLC47A*, and *TCF7L2*, have been identified (6,18,22,23). Variations in genes related to sulfonylurea metabolism, including *CYP2C9*, *ABCC8*, *KCNJ11*, *IRS1*, *CDKAL1*, *CDKN2A*, *CDKN2B*, *KCNQ1*, and *NOS1AP*, influence the effectiveness of sulfonylurea treatment (22,24). Although many studies have investigated risk alleles for diabetes and genetic variations related to diabetic drug metabolism, no specific gene markers are currently available to evaluate the development, outcome, drug treatment effect, and prognosis of diabetes.

In this study, we analyzed multiple GEO datasets to identify diagnostic biomarkers associated with immune cell infiltration in patients with diabetes. A total of 56 DEGs were identified, including 33 upregulated and 23 downregulated genes. Enrichment analyses indicated that these DEGs were mainly associated with the cytosolic ribosome, rough endoplasmic reticulum, secondary lysosome, and other pathways. Furthermore, four diagnostic markers (*B2M*, *FTL*, *SH3BGRL3*, *SOD2*) were identified using two machine-learning algorithms. Combining LASSO regression and SVM-RFE, followed by selecting overlapping genes, reduces algorithm-specific bias and improves the robustness of biomarker identification compared to single-algorithm approaches. The GSE20966 dataset was used to validate the diagnostic effectiveness of these four genes. ROC curve analysis showed that the diagnostic value of *B2M*, *FTL*, and *SOD2* for diabetes was higher than that of *SH3BGRL3*. Additionally, validation experiments confirmed that the mRNA expression of *B2M* and *FTL* is increased in the liver tissues of diabetic mice.

Beta-2 microglobulin (*B2M*) is an important subunit of major histocompatibility complex (MHC) class I and plays an important role in antigen presentation to CD8+ T cells, participating in tumorigenesis and immune regulation (25). In diabetes, increased *B2M* expression may enhance MHC class I-mediated antigen presentation on beta cells, potentially promoting cytotoxic T cell-mediated cellular damage. *B2M* is also considered a new and important urinary marker of tubular injury in early diabetic nephropathy, reinforcing its clinical relevance (26). Our finding that *B2M* expression negatively correlates with B cell infiltration and immune check-point activity further suggested a role in modulating adaptive immune responses in diabetic tissue.

Ferritin light chain (*FTL*) is associated with neurodegeneration with brain iron accumulation and is involved in lipid metabolism and autophagy (27). Dysregulation of iron metabolism is closely linked to oxidative stress and beta cell dysfunction in diabetes. Elevated *FTL* levels may be associated with increased production of reactive oxygen species and beta cell dysfunction in diabetes. Iron-laden macrophages associated with *FTL* often exhibit an M1-like pro-inflammatory phenotype, characterized by enhanced production of tumor necrosis factor-alpha (TNF-α) and IL-6, both of which aggravate insulin resistance and β-cell dysfunction (28).

*SH3BGRL3* has been reported to be closely associated with the occurrence and development of tumors such as papillary thyroid cancer, glioblastoma, urothelial carcinoma, gastric cancer, and lung adenocarcinoma (29-33). However, the relationship between these four candidate genes and the occurrence and development of diabetes has not been fully clarified.

The types of immune cell infiltration in diabetes and normal samples were assessed using ssGSEA. Different immune cell subtypes were found to be closely involved in the pathological process of diabetes. Increased infiltration of APC co-stimulation and check-point levels and decreased infiltration of CD8+ T cells, tumor-infiltrating lymphocytes (TILs), and Tregs were found to be potentially related to the occurrence and development of diabetes. In addition, *B2M* was correlated with B cells and check-point. *FTL* was correlated with neutrophils, macrophages, CD8+ T cells, cytolytic activity, Th1 cells, and proinflammatory function. *SH3BGRL3* was positively correlated with APC co-stimulation, check-point, T cell co-stimulation, CCR, and MHC class I. *SOD2* was negatively correlated with CD8+ T cells. Validation by flow cytometry or immunohistochemistry in pancreatic tissue will be conducted to confirm these patterns in future studies. The systematic exploration of correlations between the identified biomarkers and specific immune cell subtypes offers novel insight into the immunological context of these markers, which may provide future immunotherapy strategies.

Diabetes is characterized by chronic low-grade tissue and systemic inflammation, which is associated with immune cell infiltration (34). This tissue and systemic inflammation can further trigger insulin resistance. Both innate and adaptive immunity are abnormal in diabetes. The infiltration of proinflammatory M1 macrophages, CD4+ T cells, and CD8+ T cells is increased in diabetic individuals, producing various inflammatory factors that mediate inflammatory responses and insulin resistance (35-37). Moreover, the numbers of neutrophils and eosinophils are decreased in T2DM patients (38,39). Islet inflammation associated with increased proinflammatory cytokines and immune cell infiltration can lead to dysfunction of pancreatic β cells (7). Immune cells play an important role in triggering inflammation, and the mechanisms underlying impaired glucolipid metabolism in diabetes are still not completely understood. Therefore, exploring novel diagnostic biomarkers and the characteristics of immune cell infiltration in diabetes is beneficial for early diagnosis, precise treatment, and clinical prognosis. In addition, specific signaling pathway enrichment analysis of key genes and metabolic pathways associated with diabetes was also performed, providing a basis for molecular mechanism research and treatment. However, specific functional verification can be achieved by knockdown or overexpression experiments to confirm causality in the future.

Since miRNAs are short noncoding RNAs that interact with the 3′-untranslated region of mRNAs to degrade target mRNA or suppress its translation, we also explored possible miRNAs interacting with the key genes (Supplementary Figure S2). Plasma levels of miR-191 and miR-200b have been reported to be altered in people with type 2 diabetes mellitus (40). Exosomal miRNAs are pivotal regulators in the progression of diabetes, such as pancreatic β-cell injury, insulin resistance, and diabetic complications. Moreover, circulating exosomal miRNAs are potential markers for the diagnosis and prognosis of diabetes. However, the role and regulatory mechanism of miRNAs in diabetes have not been fully clarified. The analysis of possible miRNAs interacting with the key genes provides a theoretical basis for potential markers and therapeutic targets in diabetes. The simultaneous analysis of immune cell infiltration, metabolic pathway activity, and regulatory miRNA prediction provides a multidimensional view of the immunometabolic landscape in diabetes. The miRNA-mRNA interactions predicted via miRcode can be further verified by luciferase reporter gene assays.

Due to the limited sample size, clinical verification will be necessary in the future. The functions of identified biomarkers, immune cell infiltration, and the associated predictive information in diabetes were predicted by bioinformatics analysis. Further prospective studies with larger sample sizes should be conducted to verify these conclusions.

## Conclusions

DEGs were identified, and GO and KEGG enrichment analyses of these DEGs provided a research basis for understanding the specific molecular mechanisms of diabetes. The overlap between the LASSO model and the SVM-RFE algorithm was used to screen candidate genes, resulting in the identification of four key genes. Furthermore, *B2M* and *FTL* were identified based on ROC curve validation and mRNA expression analysis. The study also revealed differences in immune infiltration and immune function between diabetic and normal groups and noted that the relationship between key genes and immune infiltration in diabetes has been rarely reported. The mechanisms of key genes and immune infiltration-related factors in the diagnosis and treatment of diabetes remain to be explored. In addition, metabolic pathways between the two groups and potential miRNAs regulating the key genes in diabetes were explored to provide a foundation for further studies on the molecular mechanisms and biomarkers of diabetes.

## Data Availability

All raw data used in this study are derived from the public GEO data portal (https://www.ncbi.nlm.nih.gov/geo/; Accession numbers: GSE23561, GSE15932, and GSE20966).

## Supplementary Materials

Click to view [pdf].
