# Supplementary material for: Predicting diagnostic gene biomarkers associated with immune infiltration in patients with diabetes
Source: Braz J Med Biol Res. 2026 Sep 7;59:e15577. doi: 10.1590/1414-431X2026e15577 (PMC13557331; doi:10.1590/1414-431X2026e15577)
Supplement: Supplementary file 1 [file 1414-431X-bjmbr-59-e15577-suppl.pdf]

Figure S1. Combat algorithm correction eliminated batch effects between gene chips. **A**, Batch effect between two sets of raw gene chip data via principal component analysis (PCA). **B**, Batch effect between two sets of gene chip data after correction of the Combat algorithm.

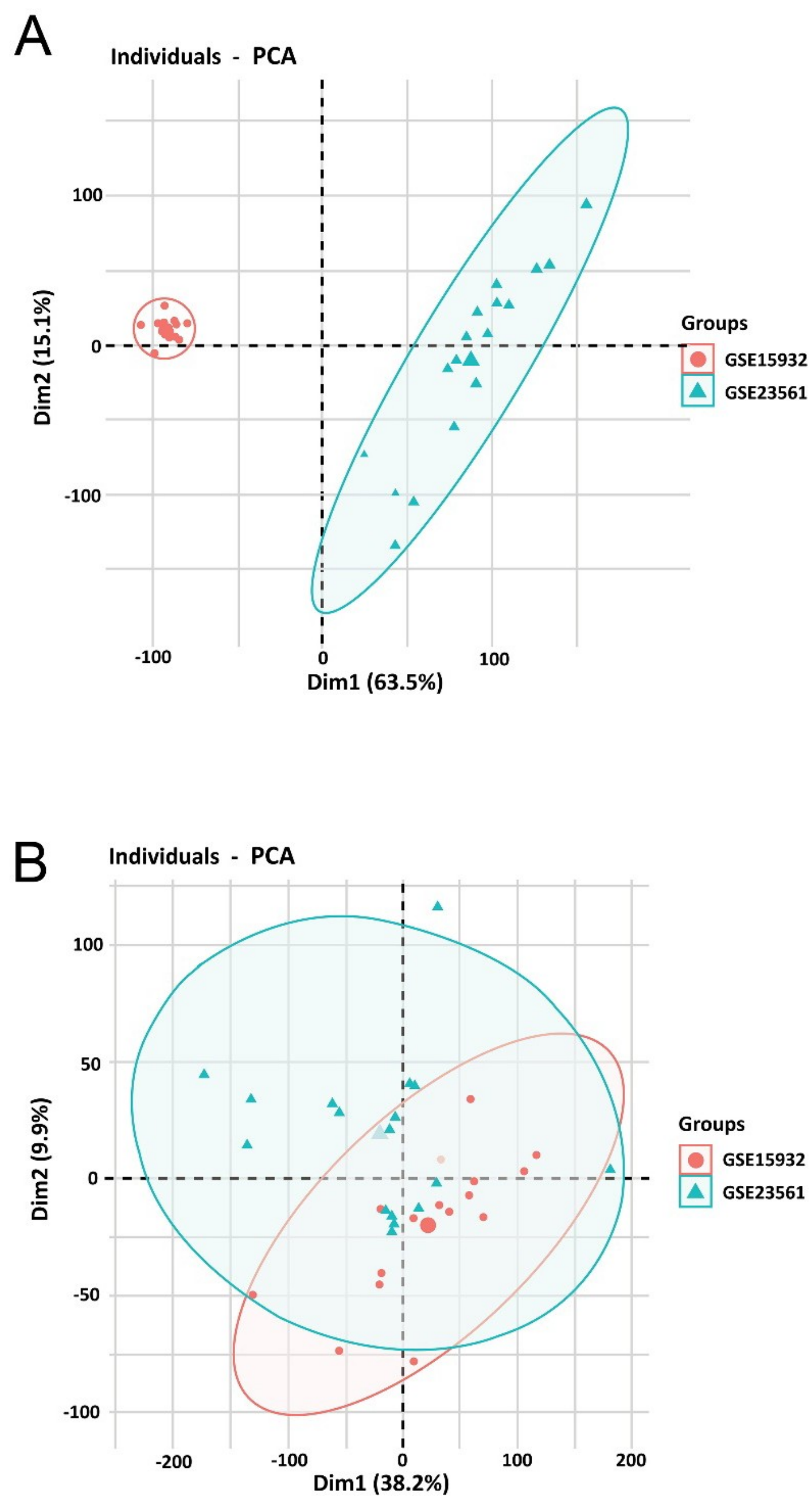

Figure S2. Possible miRNAs interacting with the key genes.

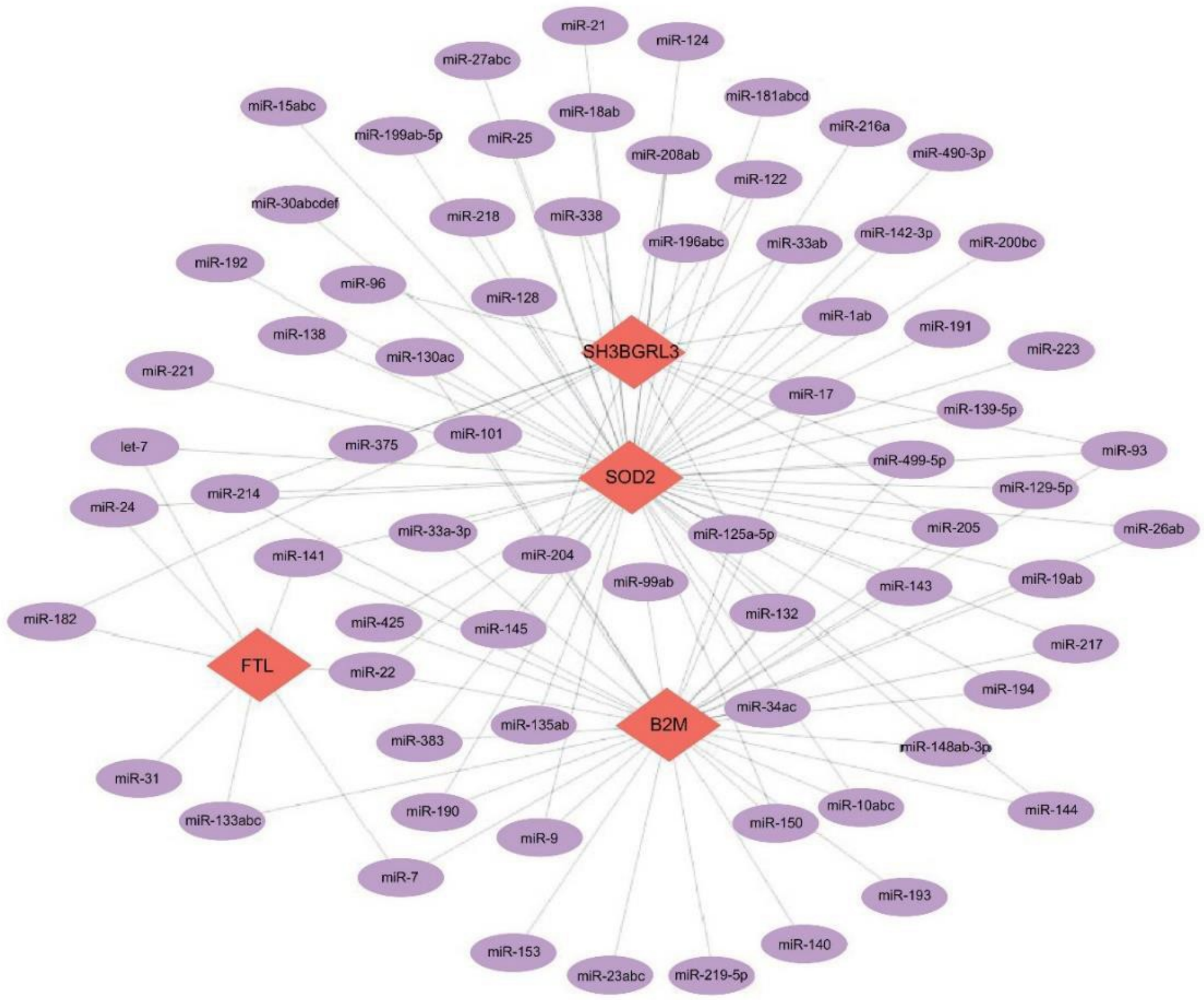

Table S1. Expression of differentially expressed genes.

**Genes**	logFC	AveExpr	*t*	P value	B
RPL7	–0.785267	3.2775388	–4.157621	0.0002243	–0.014259
RPS23	–0.766754	3.2615868	–3.819171	0.0005801	–0.934276
NCF4	0.8906784	1.8725687	3.6115347	0.001028	–1.484553
SH3BGRL3	0.7150018	2.2990564	3.4645457	0.001532	–1.866356
HLA-G	2.4977635	5.9960821	3.452051	0.0015844	–1.898488
CDX1	0.8498708	0.8461062	3.3217771	0.0022449	–2.230295
HES4	0.6368275	1.1782737	3.2999639	0.0023786	–2.285259
RPS12	–1.16129	5.9690436	–3.252598	0.0026959	–2.403996
FTL	2.0743364	7.9613715	3.0973213	0.004044	–2.787087
HBD	–1.378486	1.6289236	–3.089145	0.0041304	–2.806986
PTMA	–0.642962	2.692093	–3.037941	0.0047126	–2.930957
BTBD3	–0.621711	1.2790344	–3.035723	0.0047395	–2.9363
RPS3	–1.387291	5.53469	–2.98085	0.0054531	–3.067832
RPL19	–1.05214	4.7087965	–2.973537	0.0055556	–3.085262
B2M	–1.440726	7.5986261	–2.909507	0.0065334	–3.236816
RPS24	–0.673022	2.9225989	–2.880057	0.0070357	–3.305883
VIPR2	0.8370068	0.7593787	2.8750272	0.007125	–3.317637
RPL30	–0.66851	5.1185655	–2.849074	0.0076031	–3.378099
NCF1	0.6926565	2.3261651	2.8469248	0.007644	–3.383091
RPL11	–1.099001	5.2342629	–2.840358	0.0077703	–3.39833
CORO1A	1.0142206	3.5755617	2.8363244	0.0078489	–3.407682
UBC	0.6976474	5.5382581	2.8263095	0.0080471	–3.430865
CD52	–0.670134	1.9969363	–2.817813	0.008219	–3.450495
S100A13	0.7865041	0.7337994	2.800894	0.0085715	–3.489476
ASPSCR1	0.801735	0.8110035	2.7982464	0.0086279	–3.495564
PLOD3	0.6060014	0.88574	2.7682309	0.0092924	–3.564331
UBE2E3	–0.636071	1.1156085	–2.704377	0.010868	–3.709106
LRFN4	0.6213844	0.5286924	2.7023652	0.0109215	–3.713635
APOBEC3D	1.0465818	0.8338558	2.6664415	0.0119184	–3.794119
COX1	–3.400176	8.4276114	–2.664884	0.0119634	–3.797592
RPS29	–1.414096	5.2510662	–2.651733	0.0123504	–3.826875
HPR	0.6911149	0.8743291	2.5724759	0.0149388	–4.001376
RFNG	1.0521774	1.5079893	2.5399015	0.016141	–4.072092
PAGE5	0.73402	0.7745479	2.518667	0.016972	–4.117869
RPL27	–0.648025	5.2716939	–2.484383	0.0183971	–4.191236
CIB3	2.0090027	3.0756786	2.4653753	0.0192336	–4.231619
PRMT7	1.4264612	1.3478862	2.4652982	0.0192371	–4.231783
RPS17	–0.600192	4.4507253	–2.461477	0.0194094	–4.239876
ACADS	0.7465648	0.7991287	2.4555334	0.0196803	–4.252447
ZNF580	0.6055343	1.0024687	2.4463942	0.0201035	–4.271736
SOD2	0.814508	2.2309189	2.4346031	0.0206619	–4.296551
LATS2	0.7876777	0.9148136	2.4235962	0.0211959	–4.31964
ZDHHC8	1.8514771	5.2587371	2.4040875	0.0221733	–4.360388
CD276	0.7381184	0.6710348	2.3717306	0.0238853	–4.427467
EEF1A1	–2.464803	9.2662029	–2.33793	0.0258011	–4.49686
CPNE7	0.7749756	1.6939291	2.3116515	0.0273855	–4.550323
DTX1	–0.779823	2.1508888	–2.22073	0.0335676	–4.731947
GRB7	1.4862729	7.6554024	2.184371	0.0363713	–4.80309
S100A11	0.631171	3.3518672	2.149434	0.0392604	–4.870632
SLC17A7	0.8172056	0.9302179	2.1223816	0.0416358	–4.922372
LIM2	1.4419956	4.7813553	2.1154004	0.0422691	–4.935644
MRPL49	1.069701	0.8420713	2.0852086	0.0451074	–4.992663
RPS4X	–0.732347	4.51802	–2.081088	0.0455076	–5.000397
LYZ	–1.264712	5.0567546	–2.074806	0.0461236	–5.012165
TXNIP	–0.763159	5.1563065	–2.051537	0.04847	–5.055518
CD109	0.7998073	0.6935719	2.039964	0.0496757	–5.076942

Table S2. Clinical characteristics of the control and type 2 diabetic (T2DM) subjects in the validation cohort (GSE20966).

Characteristics	Control	T2DM
Age (years)	60 ± 5	67 ± 7
Males	6	7
Females	4	3
BMI (kg/m^2)	30.5 ± 6.5	30.9 ± 6.2
Anti-diabetes therapy	–	Oral (n=6); insulin (n=3); not available (n=1)

Age: Control *vs* T2DM, P=0.019; BMI: Control *vs* T2DM, P=0.917. The clinical characteristics presented in the table are derived from "Gene expression profiles of Beta-cell enriched tissue and obtained by laser capture microdissection from subjects with type 2 diabetes. Marselli L, Thorne J, Dahiya S, Sgroi DC, Sharma A, Bonner-Weir S, et al. PLoS One. 2010;5(7):e11499, doi: 10.1371/journal.pone.0011499.

Table S3. Correlation of the *B2M* gene with immune infiltration parameters.

Symbol	Correlation	P value
APC_co_inhibition	–0.1531	0.394984
APC_co_stimulation	–0.19586	0.273497
B_cells	–0.39873	0.022261
CCR	0.061832	0.731723
CD8+_T_cells	0.173128	0.333883
Check-point	–0.38469	0.027798
Cytolytic_activity	0.136698	0.446557
HLA	–0.0498	0.782647
Inflammation-promoting	0.118316	0.510469
Macrophages	0.022727	0.900246
MHC_class_I	0.327206	0.063573
Neutrophils	0.151404	0.398768
NK_cells	0.152933	0.395506
Parainflammation	–0.26437	0.136872
pDCs	–0.33623	0.0563
T_cell_co-inhibition	–0.28576	0.107018
T_cell_co-stimulation	–0.27039	0.127913
Th1_cells	0.069184	0.70114
TIL	0.226939	0.203304
Treg	0.216912	0.224405
Type_I_IFN_response	–0.03443	0.849018

APC: antigen presenting cell; CCR: C-C chemokine receptor; HLA: human leukocyte antigen; MHC: major histocompatibility complex; NK cells: natural killer cells; pDCs: plasmacytoid dendritic cells; TIL: tumor-infiltrating lymphocytes; IFN: interferon.

Table S4. Correlation of the *FTL* gene with immune infiltration parameters.

Symbol	Correlation	P value
APC_co_inhibition	0.205415	0.251463
APC_co_stimulation	0.232286	0.192637
B_cells	0.270053	0.128399
CCR	–0.19184	0.283593
CD8+_T_cells	–0.58155	0.00049
Check-point	0.038436	0.831584
Cytolytic_activity	–0.42112	0.015346
HLA	–0.04011	0.824343
Inflammation-promoting	–0.36297	0.03858
Macrophages	0.385695	0.027368
MHC_class_I	–0.05481	0.761306
Neutrophils	0.611297	0.000213
NK_cells	–0.33211	0.058994
Parainflammation	–0.16009	0.371983
pDCs	–0.11197	0.53357
T_cell_co-inhibition	0.225267	0.20672
T_cell_co-stimulation	–0.11029	0.539733
Th1_cells	–0.37834	0.030654
TIL	–0.32453	0.065866
Treg	–0.24733	0.164776
Type_I_IFN_response	–0.24599	0.167127

APC: antigen presenting cell; CCR: C-C chemokine receptor; HLA: human leukocyte antigen; MHC: major histocompatibility complex; NK cells: natural killer cells; pDCs: plasmacytoid dendritic cells; TIL: tumor-infiltrating lymphocytes; IFN: interferon.

Table S5. Correlation of the *SH3BGRL3* gene with immune infiltration parameters.

Symbol	Correlation	P value
APC_co_inhibition	0.015711	0.930847
APC_co_stimulation	0.495989	0.003714
B_cells	–0.07152	0.691503
CCR	–0.59893	0.000304
CD8+_T_cells	–0.21758	0.222953
Check-point	0.476939	0.005478
Cytolytic_activity	0.048797	0.786934
HLA	–0.09191	0.609696
Inflammation-promoting	–0.15842	0.377049
Macrophages	–0.13603	0.448801
MHC_class_I	–0.46658	0.006711
Neutrophils	0.085227	0.636065
NK_cells	–0.22447	0.209167
Parainflammation	–0.10996	0.540969
pDCs	0.180147	0.314411
T_cell_co-inhibition	0.189171	0.290457
T_cell_co-stimulation	0.401738	0.021203
Th1_cells	0.12266	0.494959
TIL	–0.23697	0.183635
Treg	–0.32955	0.06162
Type_I_IFN_response	–0.31918	0.070648

APC: antigen presenting cell; CCR: C-C chemokine receptor; HLA: human leukocyte antigen; MHC: major histocompatibility complex; NK cells: natural killer cells; pDCs: plasmacytoid dendritic cells; TIL: tumor-infiltrating lymphocytes; IFN: interferon.

Table S6. Correlation of the *SOD2* gene with immune infiltration parameters.

Symbol	Correlation	P value
APC_co_inhibition	0.168812	0.347669
APC_co_stimulation	0.154412	0.389372
B_cells	0.121658	0.498517
CCR	0.167781	0.34921
CD8+_T_cells	–0.42914	0.013358
Check-point	0.050468	0.779792
Cytolytic_activity	–0.14906	0.406165
HLA	0.013369	0.941528
Inflammation-promoting	–0.0996	0.579967
Macrophages	0.225267	0.20672
MHC_class_I	0.103275	0.565984
Neutrophils	0.13135	0.464683
NK_cells	–0.24285	0.173264
Parainflammation	–0.03676	0.838839
pDCs	–0.17614	0.325448
T_cell_co-inhibition	0.190174	0.28787
T_cell_co-stimulation	–0.0137	0.940051
Th1_cells	–0.06852	0.703902
TIL	0.005348	0.977039
Treg	–0.07453	0.679184
Type_I_IFN_response	–0.02005	0.912019

APC: antigen presenting cell; CCR: C-C chemokine receptor; HLA: human leukocyte antigen; MHC: major histocompatibility complex; NK cells: natural killer cells; pDCs: plasmacytoid dendritic cells; TIL: tumor-infiltrating lymphocytes; IFN: interferon.
